# Affective reactivity to upward social comparisons rather than social media use predicts increases in early adolescents’ depressive symptoms

**DOI:** 10.1038/s41598-026-58879-z

**Published:** 2026-06-21

**Authors:** Andrea Irmer, Florian Schmiedek

**Affiliations:** 1 DIPF | Leibniz Institute for Research and Information in Education, and Center for Research on Individual Development and Adaptive Education of Children at Risk (IDeA), Frankfurt am Main, Germany; 2 Goethe-University, Frankfurt am Main, Germany

**Keywords:** Social networking sites, Mental health, Individual differences, Smartphone, Health care, Psychology, Psychology

## Abstract

Youths differ substantially in how they emotionally respond to daily experiences. Repeated episodes of heightened negative affect in daily life may accumulate over time and increase vulnerability for developing depressive disorders. This study examined whether individual differences in negative affective reactivity to daily social experiences (i.e., social media use and upward social comparisons) predict changes in depressive symptoms over two weeks. Using a 14-day diary design with 200 early adolescents (103 female; ages 10–14), we assessed daily social media use, daily upward social comparisons, daily negative affect, and depressive symptoms at pre- and posttest. We employed multilevel structural equation modeling, estimating the within-person coupling between daily social media use (Model 1) or upward social comparisons (Model 2) and daily negative affect and tested whether between-person differences in the strength of this coupling predicted average negative affect and depressive symptom change. Findings revealed that a stronger within-person coupling between upward social comparisons and negative affect, but not between social media use and negative affect, was associated with an increase in depressive symptoms via elevated negative affect across the study. These results highlight the importance of capturing dynamic affective responses to daily social comparison processes when assessing psychological risk in early adolescence.

**Supplementary Information:**

The online version contains supplementary material available at 10.1038/s41598-026-58879-z.

## Introduction

Experiencing emotionally negative events is part of daily life, yet individuals differ substantially in how frequently, intensely, and persistently they experience negative affect in response to such events^1,2^. These micro-level affective processes (i.e., day-to-day fluctuations in affect as emotional responses to environmental stimuli) have been proposed to serve as building blocks of mental health or psychopathology^3,4^. Specifically, repeated episodes of heightened negative affect in daily life may accumulate over time and increase vulnerability for developing depression^5,6^, a mental disorder defined by persistent sadness, loss of interest, and emotional dysregulation^7^.

Several longitudinal studies have supported this assumption by demonstrating that both the average level of daily negative affect and individual differences in emotional reactivity to daily stressors predict mental health outcomes years later. For instance, evidence from adult samples indicates that individuals with higher average levels of daily negative affect and stronger affective reactivity to everyday stressors were more likely to develop depressive disorders a decade later^2^. Moreover, heightened emotional reactivity rather than mere exposure to stressors has been shown to predict the onset of chronic health conditions^1^. Evidence from younger populations suggests that these processes are already relevant earlier in development: Higher negative emotion reactivity to daily school-related problems has been found to be associated with greater depressive symptoms in early adolescents (i.e., fifth graders^8^. Furthermore, greater inertia of negative emotional states (i.e., more persistent negative emotional states) in early adolescence has been shown to predict the onset of depression 2.5 years later^9^.

However, what underlying processes contribute to more frequent, intense, or persistent experiences of negative mood and to heightened affective sensitivity to adverse experiences? One proposed mechanism is that greater exposure to negative events in daily life may amplify emotional responses over time. Consistent with this idea, early adolescents facing more frequent negative events showed stronger emotional responses, reflected by both elevated negative affect and diminished positive affect^5^. Importantly, early adolescents with greater cumulative stress showed even steeper declines in affect following negative events, suggesting that chronic exposure to negative events may potentially enhance affective reactivity, increasing vulnerability for long-term mental health problems^5^. Importantly, heightened affective reactivity does not necessarily imply sustained negative mood states. Individuals may respond strongly to specific daily events yet recover quickly, such that these transient reactions do not accumulate into a persistently elevated affective state^10^. Depressive symptomatology, however, is characterized less by isolated emotional spikes and more by enduring elevations in negative affect^7^. From a micro-to-macro perspective, repeated episodes of heightened reactivity may only confer risk when they translate into more frequent or prolonged experiences of negative affect across time^3,11^. Elevated average negative affect can therefore be conceptualized as the cumulative affective load through which momentary emotional reactivity becomes linked to longer-term depressive symptom development^3^. Accordingly, examining the dynamic interplay between daily experiences, affective responses, and negative affect is crucial not only for understanding current emotional functioning but also for identifying who is at risk for future mental health difficulties^3^.

The present work therefore aims to investigate which everyday experiences shape affective functioning during early adolescence, a developmental period marked by heightened emotional sensitivity and variability^12^. It thereby focuses on two types of social experiences that may be emotionally relevant during this time: social media use as a source of exposure to socially relevant information and upward social comparisons as a cognitive evaluation of social information in relation to the self. Specifically, the current study seeks to examine whether between-person differences in individuals’ affective reactivity to daily social media use and daily upward social comparisons are associated with average negative affect and change in depressive symptoms. For this aim, we focus on early adolescence (ages 10–14 years^13^ for four reasons. First, early adolescence marks the transition from childhood to adolescence^14^ and involves pronounced physical, cognitive, and social changes contributing to increased emotional intensity, heightened sensitivity to environmental influences, and significant changes in neurobiological systems involved in affect regulation^12^. These developmental characteristics likely amplify the psychological impact of daily experiences, making early adolescents particularly vulnerable to the cumulative effects of elevated negative affect and affective reactivity. Second, epidemiological studies underline this increased vulnerability, showing that many internalizing disorders, particularly depression, first emerge during this transition from childhood to adolescence^14–16^. Third, early adolescence is characterized by increasing importance of peer relationships and social status and heightened sensitivity to social evaluation^17^. As a result, how early adolescents evaluate social information in relation to the self may become particularly consequential for their emotional experiences. At the same time, engagement with social media in Germany typically begins during this developmental period^18^, providing a context in which individuals are frequently exposed to socially relevant information. And fourth, despite its theoretical importance, early adolescence remains comparatively understudied relative to middle and late adolescence, highlighting the need for further research specifically targeting this sensitive life stage^13^. However, consequently, some of the literature reviewed below is based on broader adolescent or adult samples; where relevant, we explicitly acknowledge these differences.

### Social media use and early adolescent well-being

Nowadays, youths spend a lot of time on social media platforms such as Instagram, TikTok and YouTube, where they consume social information and often respond emotionally to it^19^. Such platforms have profoundly changed individuals’ social environments by providing constant exposure to curated social information.

The intense engagement with social media has sparked concerns about potential adverse effects on mental health and has been suggested as a factor contributing to rising rates of adolescent depression and suicide in recent years^20–22^. Consequently, a growing body of empirical research has examined the relation between social media use and depressive symptoms. However, findings remain highly heterogeneous: some studies report small but significant associations between time spent on social media and depressive symptoms^23^, while others do not report meaningful links^24^. Given the limited availability of research specifically focusing on early adolescence, some of the following literature draws on broader adolescent samples. For instance, an 8-year longitudinal study found no evidence for time spent on social media to predict individual changes in adolescent depression^24^, and other research showed that access to and frequency of social media use were unrelated to later depressive symptoms^25^. However, a recent study of early adolescents aged 10 to 14 years found that problematic social media use was linked to different dimensions of depressive symptoms^26^, pointing to the importance of examining these variables specifically during this developmental window. Meta-analytic evidence with 11- to 18-year-old adolescents^27^ and across ages^28^ further supports this inconsistency, revealing a small but statistically significant average association between social media use and depressive symptoms, alongside substantial heterogeneity among studies. This heterogeneity suggests that the impact of social media use may depend on contextual and person-specific factors. It may not be the mere quantity of social media use, but rather the quality of users’ experiences – what they consume, how they interpret it, and how they react emotionally – that determines its impact on well-being^29^. Hence, it should be differentiated between mere exposure to social information (i.e., social media use) and the personal interpretation of social information. This distinction aligns with cognitive appraisal theories of emotion^30^, which posit that emotional responses are shaped not by exposure to stimuli per se, but by how individuals evaluate and interpret these stimuli.

The present work investigates the affective responses to consuming social information on Instagram, TikTok, and YouTube building on and extending prior findings using the same data set. In this previous work, we investigated within-person associations between social media use and negative affect using a 14-day diary design with early adolescents. While we found no significant average within-person effect of social media use on negative affect, we did observe substantial between-person differences in the strengths of this within-person coupling. In other words, some youths appeared emotionally sensitive to their daily social media engagement, whereas others remained largely unaffected. These interindividual differences raise the possibility that social media may still contribute to long-term mental health risks for vulnerable individuals, depending on their affective sensitivity to consuming social media content. Using the same data, the first main aim of the current study is to examine this heterogeneity more closely by testing whether early adolescents who react more strongly with negative affect to the behavioral exposure to social information (i.e., who exhibit a stronger within-person coupling between social media use and negative affect) show increases in depressive symptoms over two weeks via elevated average negative affect.

### Upward social comparisons

Upward social comparisons refer to the process of evaluating oneself in comparison to others who are perceived to be better off in at least one domain such as appearance, popularity, or competence^31,32^. In adults, upward social comparisons have been linked to heightened negative affect, diminished self-esteem, stress, and overall declines in subjective well-being^33,34^. Meta-analytic research has supported such findings, showing that upward social comparisons are associated with worsened mood^35^ and reduced mental health^36^.

Within a cognitive appraisal framework^30^, upward social comparisons can be understood as outcomes of a cognitive process through which social information is evaluated in relation to the self. During early adolescence, when peer relationships, social status, and self-evaluative concerns become increasingly important^17^, opportunities for upward social comparisons may arise in a wide range of offline and online contexts. For example, youths may compare themselves to classmates who appear more socially accepted or physically attractive, friends who seem more popular, athletic, or successful, or acquaintances perceived as having more desirable abilities or lifestyles. Social media platforms may constitute one particularly salient context in which such comparisons occur because they provide frequent access to curated and idealized social information^37^. Viewing these seemingly perfect worlds can give rise to the impression that others are happier, more attractive and popular, or generally living more fulfilling lives^38^. Supporting this idea, empirical research has documented significant associations between social media use and upward social comparisons in adults^33^ and early adolescents^38^. Furthermore, experimental research with undergraduates has shown that exposure to social media profiles high in upward comparison information (e.g., high activity social networks) leads to immediate decreases in state self-esteem^39^. Importantly, the present study focuses on upward social comparisons as a result of a cognitive appraisal of social information, regardless of what specifically triggered this impression of others living a better life. We build on prior work with the current sample, in which we found daily upward social comparisons to be associated with increased negative affect^38^. This association showed substantial between-person variability, implying that early adolescents differed in how strongly daily upward social comparisons were linked to daily negative affect. Focusing on negative affect as a precursor of depressive symptoms, the second main aim of the present work is to investigate whether youths who react more strongly with negative affect to upward social comparisons on average report higher negative affect across the study, which in turn predicts increases in depressive symptoms. This allows us to test whether the strength of the previously observed short-term association between upward social comparisons and negative affect accumulates into elevated average negative affect and might thus contribute to proximal variation in early adolescent depressive symptoms.

### The present study

The present study investigates whether variability in early adolescents’ within-person affective dynamics is associated with differences in depressive symptom development across the study period. Linking within-person dynamics to longer-term outcomes requires research designs that can capture both the occurrence of daily experiences and the variability in emotional responses to them. Intensive longitudinal designs combined with within-person modeling approaches are particularly suited to examining these dynamic emotional processes and their predictive value for mental health trajectories^40^. However, although upward social comparisons and negative affect were assessed on a daily basis, it is important to consider that such reports may reflect both situational fluctuations and more stable individual tendencies to interpreting social information or to experiencing negative mood. Accordingly, daily assessments, in general, do not necessarily capture purely state-like processes but may represent a combination of trait-like and temporally varying components.

We used data from the zEbra study, which combined pre- and posttest assessments with a 14-day diary design in a sample of 200 early adolescents aged ten to 14 years. We hypothesized that a stronger within-person coupling between daily social media use and daily negative affect is linked to heightened average negative affect across the study, which, in turn, is associated with an increase in depressive symptoms from pre- to post-test (H1, Model 1). This hypothesis assumes that individuals may differ in how they respond emotionally to behavioral exposure to social information (i.e., social media use) and that these differences may accumulate into distinct mental health trajectories.

Building on cognitive appraisal theories of emotion^30^, which emphasize the role of evaluative processes in shaping emotional responses, we further examined affective reactivity to upward social comparisons as a process of interpreting social information in relation to one’s own situation. We expected that a stronger within-person coupling between daily upward social comparisons and daily negative affect is linked to heightened average negative affect across the study, which, in turn, is associated with an increase in depressive symptoms from pre- to post-test (H2, Model 2).

This study builds on prior analyses using the same dataset^38^, which demonstrated significant average within-person associations between upward social comparisons and negative affect but non-significant average within-person associations between social media use and negative affect. Crucially, both associations showed significant heterogeneity across individuals (i.e., random effect variance). The current study extends our prior work by investigating whether between-person differences in the strength of these daily couplings – reflecting person-specific affective reactivity to social media use and upward social comparisons – predict changes in depressive symptoms over two weeks via heightened average negative affect. By capturing these day-to-day processes, this study contributes to a growing body of research highlighting how micro-level affective dynamics can accumulate into meaningful long-term outcomes. Understanding these person-specific affective pathways is essential for identifying early adolescents who may be particularly vulnerable to the adverse emotional consequences of digital engagement and social comparison processes. Importantly, the present study does not model upward social comparisons as a direct mechanism of social media use. Rather, we examine affective reactivity to two daily experiences that may co-occur^38^ but are conceptually distinguishable, namely exposure to social information and the evaluation of such information in relation to the self. In doing so, the current study provides a more nuanced understanding of how behavioral exposure to and subjective evaluation of social information may contribute to vulnerability for depression during the developmentally sensitive period of early adolescence^41^.

## Method

Data and analysis code to reproduce the results reported in this work can be found in the Open Science Framework (https://osf.io/s9xug/).

### Participants

A total of 200 early adolescents aged ten to 14 years (103 female; *M* = 11.71, *SD* = 1.02) and one parent per youth (163 mothers) participated in the study. Most youths indicated German as their native language (*n* = 160, 80.0%) and were enrolled in the academic tier of secondary school (*Gymnasium*; *n* = 151, 75.5%). At the time of the study, 84.5% of fathers and 32.5% of mothers were employed full-time, 10.0% of fathers and 56.0% of mothers were employed part-time, and 4.0% of fathers and 10.0% of mothers were unemployed. More than 40% of both mothers and fathers held a university degree, and around 10% reported a doctoral qualification, while lower educational levels were comparatively rare. The monthly net household income was under 2500€ for about 6% of the sample, between 2500€ and 3999€ for about one quarter, between 4000€ and 4999€ for about 17%, and more than 5000€ for about 40% of the sample (almost 14% preferred to not answer this item).

### Procedure

The present data were collected within the zEbra study (see https://osf.io/7a3jy/, for more information), comprising a baseline questionnaire for parents and early adolescents, a 14-day diary period, and a post questionnaire (for early adolescents only). All assessments were implemented as online questionnaires on soscisurvey.de. Participants were recruited through social media platforms, including the institutional Instagram account, as well as e-mails to schools, sports and music clubs, and word-of-mouth recommendations. Interested parents were provided with an online description of the study and could enroll themselves and their children. Participating parents first completed a questionnaire (∼10 min) gathering background information about the family, such as net household income or native language of the participating child. The next step involved the baseline assessment for youths (∼30 min), which started with an informational video explaining the study procedure and providing guidance on how to answer the items. After watching the video, youths responded to items concerning their personality and depressive symptoms, for instance. On the following day, the 14-day diary phase began. Each evening at 7 p.m., participants received an e-mail with a link to a brief online questionnaire (∼10 min) that remained accessible for three hours. They were instructed to complete the questionnaire just before bedtime. After completing the diary phase, participants received a post-study questionnaire (∼10 min), which included items similar to those in the baseline assessment (e.g., depressive symptoms), along with additional measures such as problematic social media use.

Study participation was voluntary and could be terminated at any time without giving reasons. To take part, youths had to own a smartphone with internet access and be proficient in the German language. Compensation was provided as follows: 5€ for completing the baseline questionnaire and 5€ for completing the post-study questionnaire. Additionally, participants received 1€ for each completed daily questionnaire, with an extra 10€ bonus for completing at least 12 out of 14 daily questionnaires. At the end of the study, they could receive their earned amount as a voucher. Ethical approval for the study was granted by the Ethics Committee of the DIPF | Leibniz Institute for Research and Information in Education (DIPF_EK_2021_11). Separate written informed consent was obtained from both parents (and/or legal guardian) and youths. All methods were performed in accordance with the relevant guidelines and regulations.

### Measures

#### Daily measures

Negative Affect. Participants’ negative affect was assessed using four items (“Today, I felt sad/unhappy/miserable/afraid”) rated on a 5-point scale from “*not at all true*” to “*completely true*”. This measure has been validated in previous studies^38,42^.

Social Media Use. To assess social media use, youths reported how much they had used Instagram, TikTok, and YouTube that day. Responses were given on a 5-point scale ranging from “*not at all*” to “*very much*”. Item responses were averaged to form a composite score, with higher values reflecting higher levels of social media use. This self-report measure has demonstrated good accuracy in capturing social media use^43^.

Upward Social Comparisons. Participants’ upward social comparisons were measured using six items with a common stem “Today, I had the feeling that others…” and the following endings: “have a better life than me”, “are happier than I am”, “are more popular than I am”, “are prettier than me”, “were doing more or cooler things than me”, “have more or cooler stuff than me”. Items were rated on a 5-point Likert scale from “*not at all true*” to “*completely true*”. The scale demonstrated good reliability (see Table 1) and has been used in previous research^38^.

### Person-level measures

Depressive Symptoms. Depressive symptoms were assessed at the baseline and post questionnaire. We used the Children’s Depression Screener (ChilD-S)^45^, a brief self-report measure designed to screen for depressive symptoms in children and adolescents. The scale comprises eight items measuring core symptoms of depression, such as low mood and loss of interest. To ensure consistency across the study, the original 4-point response format was adapted to a 5-point scale, aligning with the response format used for most other constructs. Higher scores indicated greater depressive symptomatology.

### Covariates

Sex. Parents reported their child’s sex and responses were coded as 0 = male, 1 = female, and 2 = non-binary. No parent indicated that their child was non-binary.

Age. Parents reported their child’s age and responses were coded as 1 = 10 years, 2 = 11 years, 3 = 12 years, 4 = 13 years, 5 = 14 years. Two participants were excluded from analyses including the age variable, because parents reported ages outside the eligible range.

### Analyses

**Fig. 1:**
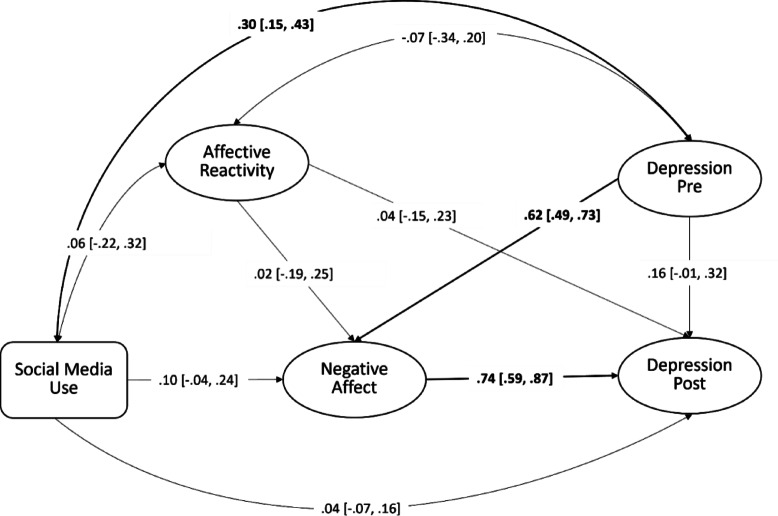
Results of model 1.

To examine whether between-person differences in within-person processes predict between-person differences in mental health outcomes, we employed two multilevel mediation models that have recently been introduced in the literature^40^. Thereby, within-person processes are modeled as the within-person coupling of daily social media use and daily negative affect (Model 1) and as the within-person coupling of daily upward social comparisons and daily negative affect (Model 2). Accordingly, affective reactivity is operationalized as a model-derived parameter reflecting these within-person associations (i.e., within-person slopes). Random effects for the within-person coupling were included, allowing for the between-person variation in strength of these couplings to be used as Level-2 predictors of average negative affect across the diary period and of depressive symptoms assessed at post-test. Thus, between-person differences in affective reactivity represent individual differences in the strength of these within-person associations. By including depressive symptoms at pre-test as a covariate and modeling depressive symptoms at post-test as the outcome, both models estimate change in depressive symptoms over time. In Model 1, average social media use (Model 2: upward social comparisons) served as another Level-2 predictor of post-test depressive symptoms. In both models, average negative affect served as a Level-2 mediator and pre-test depressive symptoms predicted average negative affect to account for the possibility that prior depressive symptomatology influenced general affect during the diary period. Furthermore, we allowed social media use (Model 2: upward social comparisons) and the within-person coupling of social media use and negative affect (Model 2: upward social comparisons and negative affect) to correlate with pre-test depressive symptoms (see Fig. 1 for a depiction of the model structure at the between-person level).

In contrast to previous applications of this approach^40^, we modeled latent variables instead of using manifest variables, with one exception: social media use, which was treated as a manifest variable. Social media use in this study refers to the mean usage of Instagram, TikTok, and YouTube, making it a formative construct rather than a reflective one. That is, modeling social media use as a latent variable would be conceptually inappropriate because the usage of individual platforms does not represent interchangeable reflections of a single construct but instead jointly define the overall social media use variable. The remaining constructs (negative affect, upward social comparisons, and depressive symptoms) were modeled as reflective latent variables, with the corresponding items specified as indicators. As social media use, upward social comparisons, and negative affect contain both within-person (Level 1) and between-person (Level 2) variance, they were decomposed into latent within-person and between-person components.

Following the procedure described previously^40^, we examined direct, indirect, and total effects of the central predictor in this work: the within-person coupling between social media use and negative affect (Model 1) or upward social comparisons and negative affect (Model 2). The direct effects capture the association between this central predictor and change in depressive symptoms, independent of the mediator. The indirect effects examine whether this association is mediated by average negative affect, while the total effects represent the combined effect of both direct and indirect pathways on change in depressive symptoms. Direct effects are reported as standardized coefficients (STDYX), whereas indirect and total effects are derived using MODEL CONSTRAINT in Mplus and correspond to combinations of unstandardized path coefficients; they are therefore reported on the unstandardized metric.

The models were estimated in M*plus* Version 8.9^46^. Bayesian estimation was conducted using two MCMC chains with a default 50% burn-in, as implemented in M*plus*. We used default, non-informative priors, 4,000 iterations, and a thinning factor of 30. We ensured convergence by inspecting trace plots, potential scale reduction factors, and autocorrelation diagnostics. Parameters were interpreted as statistically significant in case their 95% credible interval did not include zero.

## Results

### Descriptive results

2382 out of 2800 possible data points were available, implying a good study compliance rate of 85%. Table 1 shows the descriptive statistics of the variables of interest, including means, standard deviations, mean intraindividual standard deviations as well as their standard deviations, intraclass correlations, and reliabilities on within- and between-person levels. Table 2 presents the within- and between-person correlations of the study variables.

**Table 1:** Descriptive Statistics of Social Media Use, Upward Social Comparisons, and Negative Affect.

Item	M (SD)	M ISD (SD)	ICC	Reliability within/between
Social media use				
How much did you use Instagram today?	1.28 (0.72)	0.18 (0.34)	0.70	
How much did you use TikTok today?	1.65 (1.13)	0.33 (0.45)	0.75	
How much did you use YouTube today?	2.17 (1.22)	0.73 (0.37)	0.55	
Upward Social Comparisons				0.83/0.92
Today, I had the feeling that others have a better life than me.	1.56 (1.03)	0.47 (0.47)	0.59	
Today, I had the feeling that others are happier than I am.	1.57 (1.00)	0.46 (0.45)	0.61	
Today, I had the feeling that others are more popular than I am.	1.74 (1.18)	0.45 (0.46)	0.70	
Today, I had the feeling that others are prettier than me.	1.69 (1.13)	0.44 (0.47)	0.68	
Today, I had the feeling that others were doing more or cooler things than me.	1.73 (1.16)	0.50 (0.49)	0.64	
Today, I had the feeling that others have more or cooler stuff than me.	1.65 (1.08)	0.47 (0.49)	0.62	
Negative Affect				0.72/0.77
Today, I felt unhappy.	1.67 (1.03)	0.76 (0.48)	0.26	
Today, I felt sad.	1.52 (0.92)	0.65 (0.45)	0.26	
Today, I felt miserable.	1.57 (0.95)	0.68 (0.45)	0.29	
Today, I felt afraid.	1.32 (0.76)	0.43 (0.45)	0.37	

Items were presented in German. *M* = mean; *SD* = standard deviation; ICC = intraclass correlation (proportion of between-person variance to total variance); *M* ISD = mean intraindividual standard deviation. Reliability was estimated using McDonalds Omega^44^.

**Table 2:** Within- and Between-Person Correlations Among Study Variables.

	1	2	3	4	5	6	7
1 Sex							
2 Age	− 0.047	—					
3 Depression pre	0.137*	0.140	—				
4 Depression post	0.075	0.133	0.596*	—			
5 Social Media Use	0.040	0.351*	0.324*	0.287*	—	0.052	0.122*
6 Negative Affect	0.089	0.075	0.594*	0.756*	0.283*	—	0.311*
7 Upward Social Comparisons	0.190*	0.137	0.502*	0.617*	0.389*	0.674*	—

Values below the diagonal represent between-person correlations and values above the diagonal represent within-person correlations. All correlations are based on observed scale scores; in the main analyses, the corresponding constructs were modeled as latent factors (with the exception of social media use).

* = *p* <.05.

### Predicting change in depressive symptoms: multilevel mediation models

Convergence of the two Markov Chain Monte Carlo chains was achieved, as indicated by a potential scale reduction (PSR) factor of 1.003 for both models. Visual inspection of trace plots and autocorrelation plots revealed no irregularities, further supporting adequate convergence.

Results of Model 1 are illustrated in Fig. 1. Hypothesis H1 was not supported by the data. Neither higher social media use nor the strength of the within-person coupling between daily social media use and negative affect were significantly associated with higher average negative affect across the study or with change in depressive symptoms. However, higher average negative affect was linked to an increase in depressive symptoms from pre- to post-test. The indirect effect (b = 0.03 [−0.29, 0.42]) and the total effect (b = 0.12 [−0.35, 0.66]) were not significant. R^2^ values (computed by M*plus*) indicated that 44% of the variance of average negative affect and 77% of the variance of post-test depressive symptoms could be explained by the predictors included in Model 1.

**Fig. 2:**
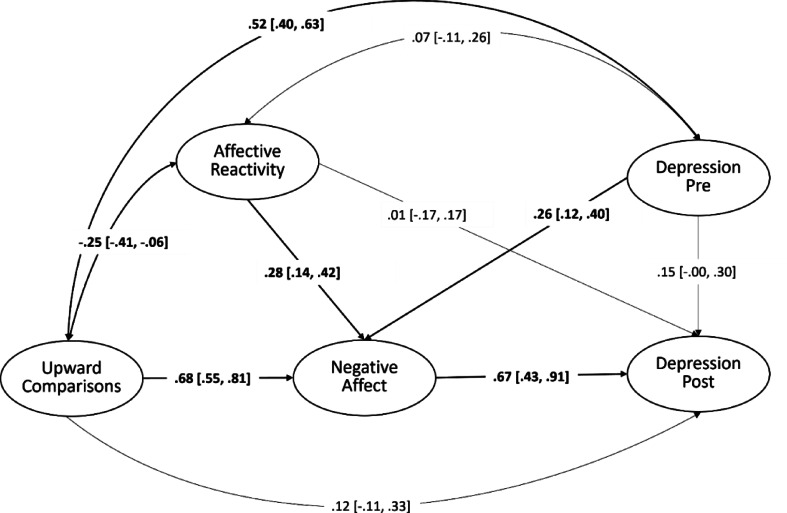
Results of model 2.

Results of Model 2 are presented in Fig. 2. Hypothesis H2 was supported by the data. Higher levels of upward social comparisons, as well as a stronger within-person coupling between daily upward social comparisons and negative affect were significantly associated with higher average negative affect across the study. While the strength of the within-person coupling was not directly related to change in depressive symptoms, higher average negative affect significantly predicted an increase in depressive symptoms from pre- to post-test. Both the indirect effect (b = 0.18 [0.08, 0.34]) and the total effect (b = 0.19 [0.05, 0.35]) were significant, supporting a mediated effect whereby the within-person coupling of daily upward social comparisons and negative affect contributed to increased depressive symptoms via elevated average negative affect. R^2^ values (computed by M*plus*) indicated that 72% of the variance of average negative affect and 77% of the variance of post-test depressive symptoms could be explained by the predictors included in Model 2.

### Additional analyses

To examine the robustness of the findings and to further clarify the associations between key constructs, we conducted additional analyses.

First, we re-ran the two main models including age and sex as covariates. In Model 1, age was significantly associated with social media use (β = 0.40 [0.27, 0.51]) and depressive symptoms at pre-test (β = 0.18 [0.02, 0.32]), indicating that older youths reported higher levels of social media use and higher levels of depressive symptoms at pre-test. The overall pattern of results remained unchanged relative to the primary analyses. The only difference concerned the association between depressive symptoms at pre-test and post-test, which reached statistical significance when covariates were included (see Supplemental Material S1).

In Model 2, age was significantly associated with upward social comparisons (β = 0.15 [0.00, 0.29]) and depressive symptoms at pre-test (β = 0.18 [0.03, 0.32]), indicating that older youths reported higher levels of upward social comparisons and higher levels of depressive symptoms at pre-test. Sex was significantly associated with upward social comparisons, indicating that females reported higher levels of upward social comparisons than males (β = 0.18 [0.03, 0.32]). The overall pattern of results remained unchanged relative to the primary analyses. The only differences concerned the association between depressive symptoms at pre-test and post-test, which reached statistical significance when covariates were included, and the correlation between upward social comparisons and affective reactivity, which was not significant when covariates were included (see Supplemental Material S2).

To address the conceptual relation between social media use and upward social comparisons, we conducted an additional analysis including both constructs in the same model. Specifically, we added the variable social media use to Model 2 (see Supplemental Material S3). This analysis was not intended to test a directional relationship, but to examine whether the significances of associations between upward social comparisons, negative affect, and depressive symptoms remain when accounting for shared variance with social media use. Social media use was positively associated with upward social comparisons, indicating that the constructs are related but not redundant. Importantly, however, the pattern of results for upward social comparisons remained unchanged: both higher levels of upward social comparisons and a stronger within-person coupling with negative affect were associated with higher average negative affect, which in turn predicted increases in depressive symptoms from pre- to post-test.

## Discussion

The present study investigated how between-person differences in within-person affective dynamics contribute to changes in depressive symptoms across two weeks. We focused on early adolescents, a population especially sensitive to external influences and to social comparisons, and particularly vulnerable to the onset of depression^16^^[,12^. As social media platforms such as Instagram, TikTok, or YouTube play an important role in the daily lives of youths, we examined negative affective reactivity to smartphone social media use, as a behavioral exposure to social information. In addition, we investigated negative affective reactivity to upward social comparisons as a potential result of the cognitive appraisal of social information in relation to the self. Prior work with this dataset showed that social media use and upward social comparisons are significantly associated at both the within-person and between-person level^38^; however, they represent conceptually distinct processes, reflecting exposure to versus evaluation of socially relevant information.

The present work demonstrated that who experienced higher average levels of negative affect across the study showed an increase in depressive symptoms from pre- to posttest. Consistent with prior empirical evidence^5^^[,9^ and dynamic conceptualizations of affective processes in the development of psychopathology^6^, these findings support the idea that repeated episodes of negative affect in daily life may accumulate over time, potentially gradually increasing vulnerability to developing depression.

However, it remains the crucial question: what daily experiences contribute to elevated or frequent negative affect in the everyday lives of early adolescents? We found neither social media use per se nor negative affective reactivity to social media use (i.e., the strengths of the within-person coupling between social media use and negative affect) to significantly predict average negative affect or depressive symptom change. Thus, while there was substantial variability in the within-person link between social media use and negative affect (though the average within-person effect was not significant^38^), this variability did not account for individual differences in average negative affect or in changes in depressive symptoms in the current sample. This was contrary to our expectations (H1) and implies that differences between youths in the negative emotional impact of social media use does not appear to translate into a differential risk for developing depressive symptoms.

The current study further investigated whether individual affective reactivity to general upward social comparisons are linked to increases in depressive symptoms via elevated negative affect in daily life. In prior work, we demonstrated that upward social comparisons are associated with daily negative affect and that individuals differed in the strength of this within-person association^38^. The current work extends these findings by showing that, in line with our expectations (H2), early adolescents who exhibited a stronger within-person coupling between daily upward social comparisons and negative affect, meaning they responded with greater unhappiness, sadness, or fear when perceiving others as more popular, happier, or more attractive, reported higher average levels of negative affect throughout the diary period. This elevated negative affect, in turn, predicted increases in depressive symptoms from pre- to posttest. Thereby, our data support a full mediation, suggesting that heightened negative emotional reactivity to upward social comparisons contributes to depressive symptom change through elevated daily negative affect. Hence, the current study provides evidence that early adolescents differ in their negative affective sensitivity to the impression that others are living better lives and that this variability is predictive of change in mental health across two weeks. This finding is broadly consistent with process-oriented frameworks, which posit that recurring affective states may constitute proximal mechanisms linking everyday experiences to mental health outcomes^3^. However, the present results should not be taken as evidence for purely dynamic, context-specific processes. Although upward social comparisons were assessed daily, the measure likely captures both stable individual differences (e.g., a general tendency to engage in or be affected by such comparisons) and within-person fluctuations across days.

Taking together, the current findings highlight that research should move beyond focusing only on adolescents’ affective sensitivity to social information exposure and instead consider how socially relevant information is cognitively interpreted and evaluated. From a cognitive appraisal perspective^30^, the findings suggest that emotional and depressive outcomes may be more closely linked to how early adolescents affectively respond to the evaluation of social information in relation to the self (e.g., by perceiving that others are better off than oneself) than to exposure itself (e.g., consuming the portrayal of others’ lives on social media). The additional model showed that social media use was positively associated with upward social comparisons, indicating that these constructs are related but not redundant. This finding is consistent with the idea that social media may represent one context in which upward social comparisons arise^33^^[,38^. Importantly, however, the associations between upward social comparisons, negative affect, and depressive symptoms remained unchanged when social media use was included in the model, suggesting that upward social comparisons capture an evaluative process that is not reducible to the quantity of social media use. Hence, independent of whether the impression that others are better off is shaped by social media environments, school contexts, peer interactions, or other everyday situations, the current study suggests that it is early adolescents’ negative emotional reactivity to such comparison experiences that appears relevant for proximal variation in depressive symptoms. Targeting this subgroup of individuals with a strong such response through early, tailored interventions may help reduce emotional sensitivity to social comparison processes and strengthen protective factors, buffering against the onset of depressive symptoms. Such interventions could incorporate strategies aimed at fostering self-compassion^47^^[,48^, enhancing media literacy^49^, or strengthening emotion regulation skills^50^. However, the current findings also suggest that there are early adolescents for whom the impression that others are living more fulfilling lives is not associated with an increase in depressive symptoms via stronger feelings of sadness or unhappiness. This is in accordance with social comparison theory, which proposes that upward social comparisons can have both adverse and beneficial effects depending on how individuals interpret these comparisons^31^^[,32^. Some adolescents may experience upward social comparisons as motivating or inspiring^51^^[,52^. For example, getting the impression that others are doing more exciting things or have a more athletic body may elicit aspirations, promote goal setting, and ultimately contribute to enhanced well-being for these individuals. Future research should explore whether some adolescents may even show positive affective responses - such as inspiration or motivation - when engaging in upward social comparisons, and whether these patterns are related to improved long-term outcomes. Identifying the conditions under which upward social comparisons are experienced as either threatening or inspiring could help clarify the mixed findings in the literature and inform more targeted, personalized intervention programs.

### Limitations

Several limitations should be acknowledged. First, the generalizability of the findings is limited due to the use of a convenience sample of early adolescents living in Germany. The sample was positively selected, with an overrepresentation of families with higher educational backgrounds and household incomes. Moreover, cultural differences in norms of self-evaluation and social comparison may further shape how individuals interpret socially relevant information, highlighting the importance of cross-cultural research in this domain. Future studies should therefore examine these processes in more socioeconomically and culturally diverse samples.

The second limitation concerns the use of chronological age as a proxy for developmental processes. Early adolescence is characterized by substantial variability in pubertal maturation, and youths of the same age may differ markedly in their biological and psychosocial development^17^^[,53^. Such differences may be relevant for sensitivity to social evaluation, engagement in social comparisons, and affective responses to daily experiences. As pubertal status was not assessed in the present study, we cannot determine whether individual differences in developmental timing contributed to the observed associations. Future research would benefit from including direct measures of pubertal development to more precisely capture developmental heterogeneity and to examine potential interactions with sex and other individual characteristics.

Third, social media use was measured based on the average use of Instagram, TikTok, and YouTube. While these are popular platforms among youths, the findings may not generalize to other platforms with different social or content dynamics (e.g., Snapchat, WhatsApp).

Fourth, it is important to consider that mediation models do not establish causality per se; rather, researchers specify the presumed causal direction of associations based on theory and prior empirical evidence. In the present study, affective reactivity and average negative affect were both derived from the same diary period, limiting the extent to which temporal ordering between these processes can be established. Therefore, the observed indirect effects should be interpreted as statistical associations consistent with the proposed theoretical framework rather than as direct evidence of causal mediation. Experimental and longer-term longitudinal designs are necessary to more conclusively test causal mechanisms and temporal dynamics.

The fifth limitation concerns the temporal scope of the study and the interpretation of change in depressive symptoms. In our study, depressive symptoms were assessed at days before and after the 14-day diary period. However, depressive symptomatology is generally considered relatively enduring and slow-moving, raising the question of whether meaningful change can be expected over such a limited time frame. Importantly, our aim was not to capture long-term clinical trajectories of depression per se, but rather to test a theoretically informed micro-to-macro pathway: whether stable between-person differences in daily affective reactivity accumulate into short-term shifts in symptomatology. From this perspective, the post-assessment of depressive symptoms reflects an early, proximal manifestation of vulnerability rather than a fully developed long-term outcome. Nevertheless, the short follow-up window limits conclusions about sustained change and longer-term mental health trajectories. Although our findings revealed significant changes in symptomatology over this short time frame, it is plausible that the effects of daily affective reactivity on depressive symptoms unfold more clearly over extended time frames (e.g., months or years), potentially yielding stronger and more clinically meaningful associations. Future studies should therefore combine intensive daily assessments with longer follow-up intervals to directly examine whether micro-level affective processes translate into enduring changes in depressive symptoms.

Finally, it is important to note that upward social comparisons were assessed as a general daily experience and were not explicitly tied to social media contexts. Although prior analyses with the same dataset demonstrated that daily social media use and daily upward social comparisons are positively associated at between- and within-person levels^38^, the present study does not permit conclusions about whether the comparison experiences were specifically elicited by social media use. Furthermore, because the measure was not tied to specific situations, it is not possible to fully disentangle stable individual tendencies from momentary experiences. Future research would benefit from assessing upward social comparisons in a context-specific manner (e.g., distinguishing between digital and offline comparison experiences) or event-based assessments to examine whether affective reactivity to upward social comparisons varies as a function of the context in which such comparisons are elicited.

## Conclusion

The present study contributes to a more process-oriented understanding of emotional vulnerability in early adolescence by highlighting the importance of everyday affective processes and interindividual differences in emotional reactivity. Rather than focusing solely on exposure to socially relevant experiences (i.e., social media use), the findings underscore the role of how adolescents emotionally respond to the perception that others are better off their daily lives. In line with theoretical perspectives emphasizing the relevance of affect dynamics and everyday emotional processes for the development of psychopathology^3^, the present findings suggest that repeated negative emotional responses to upward social comparisons may represent a relevant affective vulnerability process during early adolescence. Specifically, youths who reacted more strongly with negative affect when perceiving others as better off appeared more likely to show increases in depressive symptoms via heightened average negative affect in daily life. By contrast, we did not find affective reactivity to social media use to be associated with daily negative affect or with change in depressive symptoms. Hence, the current study highlights the value of distinguishing between behavioral exposure to socially relevant information (i.e., social media use) and the evaluative outcomes of interpreting social information in relation to the self (i.e., upward social comparisons) when examining the affective vulnerability processes associated with early adolescent mental health.

## Supplementary Information

Below is the link to the electronic supplementary material.

Supplementary Material 1

## Data Availability

The data that support the findings of this study are openly available in the OSF at: [ https://osf.io/s9xug/ ].
