# Supplementary material for: Affective reactivity to upward social comparisons rather than social media use predicts increases in early adolescents’ depressive symptoms
Source: Sci Rep. 2026 Jun 21;16:19189. doi: 10.1038/s41598-026-58879-z (PMC13284303; doi:10.1038/s41598-026-58879-z)
Supplement: Supplementary file 1 — Supplementary Material 1 [file 41598_2026_58879_MOESM1_ESM.docx]

**Figure S1**

*Results of Model 1 Including Sex and Age as Covariates*


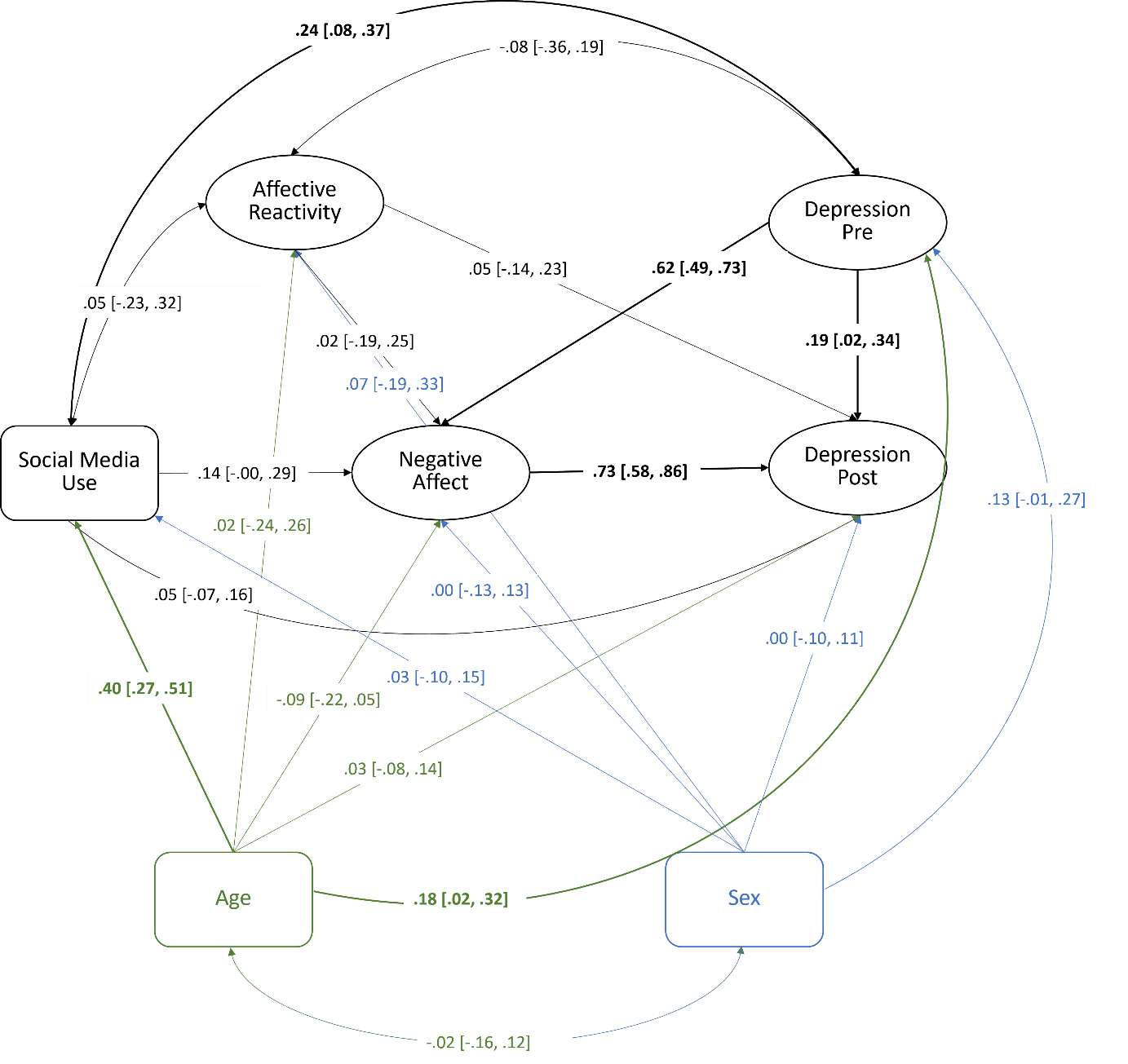


*Note*. Figure depicts standardized estimates, with 95% credible intervals in brackets. Bold text and arrows illustrate significant effects. Double-headed arrows denote correlations, whereas single-headed arrows represent directed paths. Oval shapes indicate latent variable modeling, while the rectangle indicates manifest variable modeling. Covariates are displayed in color to improve visual differentiation from the primary model paths. The potential scale reduction (PSR) factor of the model was 1.005.

The indirect effect (b = .03 [-.29, .40]) and the total effect (b = .12 [-.31, .65]) were not significant.

**Figure S2**

*Results of Model 2 Including Sex and Age as Covariates*


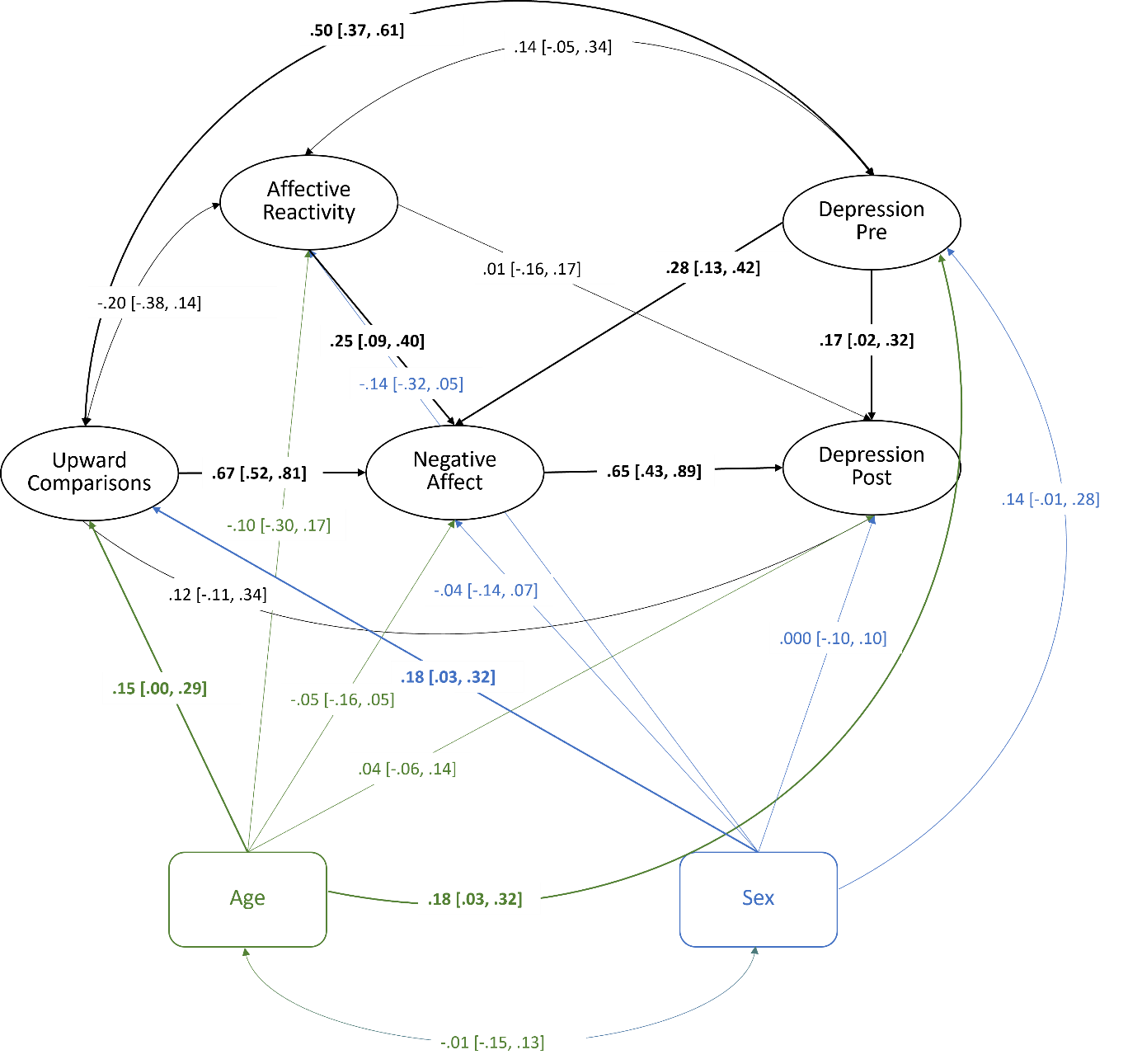


*Note*. Figure depicts standardized estimates, with 95% credible intervals in brackets. Bold text and arrows illustrate significant effects. Double-headed arrows denote correlations, whereas single-headed arrows represent directed paths. Oval shapes indicate latent variable modeling, while the rectangle indicates manifest variable modeling. Covariates are displayed in color to improve visual differentiation from the primary model paths. The potential scale reduction (PSR) factor of the model was 1.062.

The indirect effect (b = .15 [.05, .30]) and the total effect (b = .16 [.02, .34]) were significant.

**Figure S3**

*Results of An Additional Model Simultaneously Including Social Media Use and Upward Social Comparisons*

**
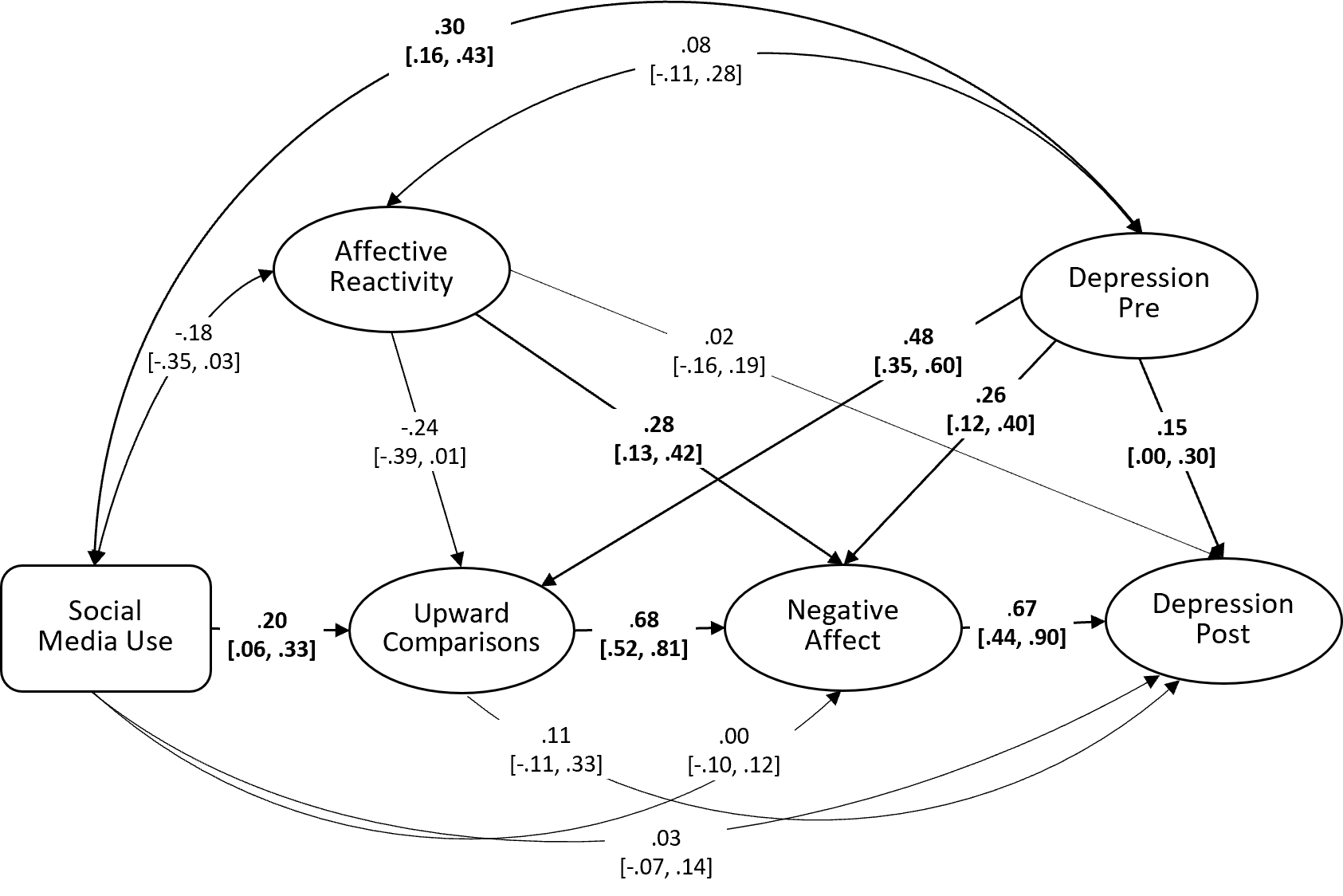
**

*Note*. Figure depicts standardized estimates, with 95% credible intervals in brackets. Bold text and arrows illustrate significant effects. Double-headed arrows denote correlations, whereas single-headed arrows represent directed paths. Oval shapes indicate latent variable modeling, while the rectangle indicates manifest variable modeling. The potential scale reduction (PSR) factor of the model was 1.013.

The indirect effect (b = .18 [.07, .35]) and the total effect (b = .20 [.05, .40]) were significant.
